# Closed Genome Sequence of a Salmonella enterica Serovar Bovismorbificans Strain Isolated from Dried Pork Sausage Associated with an Outbreak in France

**DOI:** 10.1128/MRA.00662-21

**Published:** 2021-10-07

**Authors:** Sabine Delannoy, Sabrina Cadel-Six, Laetitia Bonifait, Maï-Lan Tran, Emeline Cherchame, Louise Baugé, Karol Romero, Sandra Rouxel, Amandine Thépault, Christophe Cordevant, Marianne Chemaly, Anne Brisabois, Patrick Fach

**Affiliations:** a Anses, Laboratory for Food Safety, IdentyPath Genomics Platform, Maisons-Alfort, France; b Anses, Laboratory for Food Safety, Salmonella and Listeria Unit, Maisons-Alfort, France; c Anses, Ploufragan-Plouzané-Niort Laboratory, Unit of Hygiene and Quality of Poultry and Pork Products, Ploufragan, France; d Anses, Strategy and Programmes Department, Research and Reference Division, Maisons-Alfort, France; University of Maryland School of Medicine

## Abstract

We report here the closed genome sequence of one Salmonella enterica subsp. *enterica* serovar Bovismorbificans strain isolated from dried pork sausage consumed by a patient suffering from salmonellosis.

## ANNOUNCEMENT

Salmonella enterica subsp. *enterica* serovar Bovismorbificans is responsible for an increased number of foodborne infections in Europe (1–3). We provide here the whole-genome sequence of *S.* Bovismorbificans strain 2020LSAL11867, which has been isolated (using the ISO6579-1:2017 method) from dried pork sausage associated with a Salmonella outbreak in France. The whole genome was sequenced using NovaSeq (Illumina, Inc., San Diego, CA, USA) and MinION (Nanopore, Oxford Science Park, Oxford, UK) technologies. Prior to genomic DNA isolation for Illumina and MinION sequencing, the strain was cultivated overnight at 37°C in brain heart infusion (BHI). Genomic DNA was prepared from 2 ml of BHI overnight cultures using the Wizard high-molecular-weight (HMW) DNA extraction kit (Promega, France) according to the manufacturer’s instructions. The Illumina paired-end sequencing (2 × 150-bp format) was performed as described (4). For MinION sequencing, the DNA was neither sheared nor size selected. The MinION library was prepared with 500 ng DNA using the ligation sequencing kit (SQK-LSK109; Oxford Nanopore Technologies, France) according to the manufacturer’s instructions, and long-read sequencing was performed using a Flongle (R9.4.1) flow cell on the Oxford Nanopore MinION sequencer for 24 h.

Default parameters were used for all software unless otherwise specified. The Illumina sequencing resulted in 8,856,638 raw reads, which were analyzed for quality control, normalization, and assembly using the ARTwork workflow (5). The raw reads were normalized (100×) using BBMap v38.22 and BBNorm v36.14 (6). The reads were trimmed (Phred score, 30×; trailing, >20; minimum length, 50 bp) using Trimmomatic v0.33 (7) and *de novo* assembled using SPAdes v3.13.0 (8) (minimum contig length, 200 bp), producing 15 contigs.

The raw fast5 files produced by the MinION sequencing were base called using the ONT Guppy v4.4.2 base caller with high accuracy mode. The sequencing output was 55,329 reads. The quality of the MinION read files as determined using NanoPlot v1.28.2 (9) passed the standard quality checks with a mean read quality of 11.0 and median read quality of 11.7. The read length *N*_50_ value was 20.9 kbp.

Hybrid assembly was performed using the Unicycler v0.4.8 assembler (10), combining the sequence data sets generated by both Illumina and Oxford Nanopore Technologies sequencing. Unicycler default parameters were used, except –min_fasta_length 1000, to obtain a genome of 4,676,102 bp with a GC content of 52.29%, divided into 3 circular contigs—the chromosome (4,667,486 bp) and two plasmids of 4,712 bp (GC content, 51.10%; GenBank accession number CP073716.1) and 3,904 bp (GC content, 51.10%; CP073717.1). Unicycler was used to circularize the replicons and search for *dnaA* or *repA* alleles. If such a sequence was found, the replicon as rotated so that it began with that gene encoded on the forward strand. *S.* Bovismorbificans was attributed using the SeqSero v1.2 application on the Center for Genomic Epidemiology website (https://cge.cbs.dtu.dk/services), and the multilocus sequence type (ST) was found to be ST142. The sequence was annotated using the National Center for Biotechnology Information (NCBI) Prokaryotic Genome Annotation Pipeline (PGAP) at http://www.ncbi.nlm.nih.gov/genome/annotation_prok. Sequencing metrics are provided in Table 1.

**TABLE 1:** NCBI accession numbers and assembly metrics of the *S.* Bovismorbificans draft genome sequence

Feature	Data
GenBank accession no.	GCF_018340585.1
SRA accession no.	PRJNA722181
Origin	Dried pork sausage
Yr	2020
No. of contigs	3
Genome size (Mbp)	4.6675
GC content (%)	52.29
*N*_50_ (kbp)	4,667
No. of coding sequences (per PGAP)	4,308

### Data availability.

The genome of this strain, which harbors 4,308 coding sequences, was deposited in NCBI/DDBJ/ENA/GenBank under the accession number GCF_018340585.1. The raw data were deposited in the SRA database under the accession number PRJNA722181.
